# Structural and spectroscopic characterisation of a heme peroxidase from sorghum

**DOI:** 10.1007/s00775-015-1313-z

**Published:** 2015-12-14

**Authors:** Chukwudi I. Nnamchi, Gary Parkin, Igor Efimov, Jaswir Basran, Hanna Kwon, Dimitri A. Svistunenko, Jon Agirre, Bartholomew N. Okolo, Anene Moneke, Bennett C. Nwanguma, Peter C. E. Moody, Emma L. Raven

**Affiliations:** Department of Microbiology, University of Nigeria, Nsukka, Nigeria; Department of Chemistry, University of Leicester, University Road, Leicester, LE1 7RH UK; Department of Molecular and Cell Biology, Henry Wellcome Laboratory for Structural Biology, University of Leicester, Lancaster Road, Leicester, LE1 9HN UK; School of Biological Sciences, University of Essex, Wivenhoe Park, Colchester, CO4 3SQ UK; York Structural Biology Laboratory, Department of Chemistry, The University of York, Heslington, York, YO10 5DD UK; Department of Biochemistry, University of Nigeria, Nsukka, Nigeria

**Keywords:** Heme, Porphyrin, X-ray crystallography, Sorghum, Peroxidase, Calcium

## Abstract

A cationic class III peroxidase from *Sorghum bicolor* was purified to homogeneity. The enzyme contains a high-spin heme, as evidenced by UV–visible spectroscopy and EPR. Steady state oxidation of guaiacol was demonstrated and the enzyme was shown to have higher activity in the presence of calcium ions. A Fe^III^/Fe^II^ reduction potential of −266 mV vs NHE was determined. Stopped-flow experiments with H_2_O_2_ showed formation of a typical peroxidase Compound I species, which converts to Compound II in the presence of calcium. A crystal structure of the enzyme is reported, the first for a sorghum peroxidase. The structure reveals an active site that is analogous to those for other class I heme peroxidase, and a substrate binding site (assigned as arising from binding of indole-3-acetic acid) at the γ-heme edge. Metal binding sites are observed in the structure on the distal (assigned as a Na^+^ ion) and proximal (assigned as a Ca^2+^) sides of the heme, which is consistent with the Ca^2+^-dependence of the steady state and pre-steady state kinetics. It is probably the case that the structural integrity (and, thus, the catalytic activity) of the sorghum enzyme is dependent on metal ion incorporation at these positions.

**Electronic supplementary material:**

The online version of this article (doi:10.1007/s00775-015-1313-z) contains supplementary material, which is available to authorized users.

## Introduction

Sorghum (*Sorghum bicolor*) is an important worldwide cereal whose entire genome has recently been sequenced [1]. After wheat, maize, barley and rice, *Sorghum* is one of the world’s most important cereals; it is the most extensively grown cereal grain in Nigeria and is a widely used raw material in the brewing industry. Interest in peroxidases from sorghum arises from their potential biotechnological applications. However, there is a relatively limited amount of information on sorghum peroxidases. A peroxidase was reported in crude sorghum grain extract in 1971 [2], but with very limited biochemical characterization. Later studies reported partial purification of a peroxidase from malting sorghum [3, 4]. The most detailed characterization so far of a peroxidase from sorghum was in 2006 [5].

There are some key features of sorghum peroxidase which are not yet established—in particular, there is no information on the reactivity of the enzyme with hydrogen peroxide, there is limited information on the Ca^2+^-dependency of the enzyme, and there is no structural information at all. In the present work, we report the purification of a sorghum peroxidase from a yellow sorghum grain (SK 5912) that is widely used in brewing. We have characterised the function and reactivity of the enzyme using spectroscopic, kinetic and electrochemical methods. We also report the crystal structure of this enzyme—the first for a sorghum peroxidase—and we compare this with the known features of other class III peroxidase enzymes.

## Materials and methods

### Protein isolation and purification

Since there is no recombinant expression system available for the enzyme, it was not possible to isolate the enzyme from *E. coli* in the normal ways. Instead, the enzyme was isolated directly from sorghum grains that Dr. Nnamchi provided from Nigeria.

Sorghum grains (*Sorghum bicolor* L. Moench variety SK 5912) grown in 2009 and purchased from the Institute for Agricultural Research of the Ahmadu Bello University Zaria, Nigeria, were used. The grains were manually sorted to remove broken kernels and foreign materials before being surface sterilized by immersion in 1 % (v/v) hypochlorite solution [6]. After thoroughly rinsing with tap and distilled water, the grains were spread on a clean surface layered with soft absorbent paper and allowed to dry at room temperature overnight. Grains were crushed to a fine powder by violent bashing over an extended period in a mortar and pestle, and the protein extracted using a modified method of Nwanguma and Eze [7]. Sorghum flour (0.2 g/mL) was incubated with 100 mM sodium phosphate buffer (pH 6.0) for 30 min at 4 °C and then centrifuged at the same temperature for 30 min at 5000 rpm using a SLC 6000 Sorvall Evolution centrifuge. The protein from the supernatant was precipitated using ammonium sulphate (to 30 %, 60 % and finally 90 % saturation) on ice and with continuous stirring; the solution was centrifuged (as described above) once each saturation point had been reached and assayed for peroxidase activity (see below). The precipitate after centrifugation was redissolved in 50 mM phosphate buffer pH 8.5 and dialysed against the same buffer in three 4-hour periods and used for further purification.

For purification, a 2.5 × 20 cm column was packed with DE-52 DEAE cellulose (Whatman International Ltd, Maidstone, England) and equilibrated with 50 mM phosphate buffer, pH 8.5. The dialysed crude enzyme from the 60–90 % ammonium sulphate cut, which showed the highest peroxidase activity, was loaded onto the column and the column washed with equilibration buffer at a flow rate of 150 mL/h. The peroxidase did not bind to the DE-52 resin and was eluted during this step. Fractions (10 mL) were collected and the absorbance of the fractions monitored at 280 nm and assayed for peroxidase activity (see below). Fractions containing peroxidase activity were pooled and concentrated by centrifugation at 4000 rpm using a 10 kDa molecular weight cut-off membrane in a SLC 6000 Sorvall Evolution Centrifuge. The concentrated protein was dialysed against 50 mM phosphate buffer, pH 6.0, and loaded onto a 2.5 × 15 cm CM-52 carboxymethyl cellulose (Whatman) column equilibrated with 50 mM phosphate buffer, pH 6.0 (equilibration buffer). The column was washed with equilibration buffer using a flow rate of 90 mL/h. The bound protein was eluted using a linear gradient of 0–0.6 M NaCl (200 mL, in equilibration buffer) at the same flow rate. Small (5.0 mL) fractions were collected and monitored for peroxidase activity. Fractions containing sorghum peroxidase were pooled and concentrated (ca. 5 mL) using a 10 kDa molecular weight cut-off membrane and loaded onto a Sephadex G-75 column that had been previously equilibrated with equilibration buffer. The column was run at a flow rate 0.5 mL/min and eluted fractions were assayed for peroxidase activity and protein purity assessed by SDS-PAGE. Heme concentrations were determined using the pyridine hemochromogen assay [8]. Mass spectrometry using both MALDI-TOF and ESI (data not shown) revealed a molecular mass for the protein of 35,571 and 35,647 Da, respectively, although other peaks in the 35.5 kDa range were also present in both mass spectrometry experiments indicating that the enzyme as isolated comprises several different isoforms (probably differing in their glycosylation).

### Electronic spectroscopy

All absorbance spectra and equilibrium ligand binding experiments were measured in 100 mM sodium phosphate buffer, pH 6.0, at 25.0 °C using a Jasco V630 UV–VIS spectrophotometer. Ferrous sorghum peroxidase was generated by addition of sodium dithionite to the ferric enzyme and the ferrous-CO derivative was produced by direct bubbling of CO gas through the dithionite-reduced sample. Equilibrium binding constants, *K*_D_, were determined according to published procedures [9] and involved addition of small volumes (0.5–2.0 μL) of ligand (from an appropriate stock solution) to the protein (~2–4 μM) until no further spectral change occurred.

### Steady state activities

Steady state kinetic assays were performed in 50 mM sodium acetate buffer (pH 5.5) at 25 °C. The initial rate of oxidation of guaiacol was monitored at 470 nm in the absence and presence of calcium chloride (0.5 mM) (*ε*_470_ = 22.6 mM^−1^ cm^−1^). All reactions contained 10 nM sorghum peroxidase and were initiated by the addition of 100 μM H_2_O_2_. Values for *K*_m_ and *k*_cat_ parameters were determined by fitting the data to the Michaelis–Menten equation using the GraphPad Prism 6 software package.

### Transient state kinetics

Transient state kinetic measurements were performed using an SX.18MV stopped-flow spectrophotometer (Applied Photophysics, UK) fitted with a Neslab RTE-200 circulating water bath (25.0 ± 0.1 °C). Multiple wavelength absorption studies were carried using a photodiode array detector in 50 mM sodium acetate buffer, pH 5.5. 1.7 μM Sorghum peroxidase was rapidly mixed with 50 μM H_2_O_2_ and the spectral changes monitored over an appropriate time period; the experiment was repeated in the presence of 1 mM calcium chloride. Spectral deconvolution was performed by global analysis and numerical integration methods using Pro-Kineticist software.

### EPR spectroscopy

Aliquots of sorghum peroxidase (sodium phosphate buffer, pH 6) were frozen in Wilmad SQ EPR tubes (Wilmad LabGlass, Vineland, NJ, USA) in methanol kept on dry ice. The EPR spectra of the frozen samples were recorded at 10 K on a Bruker EMX spectrometer (X-band) equipped with a spherical high-quality ER 4122 SP9703 resonator and an Oxford Instruments liquid helium system.

### Reduction potential determination

Determination of the ferric/ferrous reduction potential (50 mM potassium phosphate buffer, pH 7.0) was using the xanthine/xanthine oxidase method, as described previously [10]. The method allows the determination of the reduction potentials from equilibrium concentrations in the presence of a suitable dye and without the need for measuring a potential; it thus avoids some of the difficulties associated with other electrochemical methods for the determinations of reduction potentials in proteins, because equilibria are achieved rapidly and there is no interference from surface contamination of electrodes [10]. Potassium phosphate buffer was made anaerobic by addition of glucose (5 mM), glucose oxidase (50 µg/ml) and catalase (5 µg/ml). A dye of known potential, in this case phenosafranin (*E*^*o*^′ = −252 mV [11]), is used and the assay mixture also included xanthine (300 µM), xanthine oxidase (50 nM), benzyl viologen (0.2 µM), and enzyme (20 µM). Changes in absorbance corresponding to the reduction of heme were measured at the isosbestic point of phenosafranin (407 nm). Reduction of the dye was measured at 520 nm, where the change caused by heme reduction was negligible. Using this method, linear Nernst plots for one-electron reduction of heme [25 mV ln(*D*_ox_*/D*_red_)], and the two-electron reduction of dye [12.5 mV ln(*D*_ox_*/D*_red_)], where *E*_ox_*, E*_red_ and *D*_ox_*, D*_red_ are the concentrations of oxidized (ox) and reduced (red) forms of enzyme (*E*) and dye (*D*), respectively, produced the expected slope (slope = 1) across a wide range of potentials, and the intercept gives a reliable value for ∆*E*^*o*^′ versus NHE (normal hydrogen electrode) with an error margin of ±2 mV (according to [10, 12]).

### Crystallography

The sorghum peroxidase crystals were grown using the sitting drop vapour diffusion technique at 291 K. The crystallisation drops were set up by mixing 2 µl enzyme (7 mg/ml) in 100 mM sodium phosphate pH 6.0 with 2 µl reservoir solution (4 M sodium formate). The crystals were prepared for X-ray diffraction by brief immersion in the reservoir solution containing 10 % glycerol for cryo-protection followed by flash-freezing in liquid nitrogen.

X-ray diffraction data were collected at Diamond Light Source on I03 beam line to 1.27Å. 1030 images of 0.1 degree were collected with an exposure time of 0.2 s using 0.9763 Å radiation. Data were processed and scaled with XDS [13] and Aimless software from CCP4 program suite [14]. The structure was determined by molecular replacement [15] using the barley peroxidase structure (PDB code: 1BGP [16]) as the search model (sequence identity 69 %, sequence similarity 82 % to BP1) and refined with REFMAC5 [17]. *N*-glycans pyranosides were checked for stereochemical errors and validated against omit mFo-DFc density with the Privateer software [18] from the CCP4 suite [14]. Data collection and refinement statistics are shown in Table 1. The crystals have space group P3_1_21 with unit cell dimensions of *a* = *b* = 58.62 Å, *c* = 208.41 Å, *α* = *β* = 90°, *γ* = 120°, and contain one molecule in the asymmetric unit. The co-ordinates and diffraction data have been deposited with PDB ID 5OAG.

**Table 1:** Data collection and refinement statistics

Space group	P3_1_21
Unit cell (Å)	58.62, 58.62, 208.41
Data collection statistics*	
Resolution (Å)	36.36–1.27 (5.7–1.27)
*R*_merge_	0.033 (0.704)
*I*/*σI*	19.4 (2.1)
Completeness (%)	98.4 (93.9)
Redundancy	5.4 (4.9)
Wilson B value (Å^2^)	18.8
Refinement	
Resolution (Å)	36.36–1.27
No. of reflections	103,499
No. of reflections for R_free_	5440
*R*/*R*_free_ (%)	13.61/15.93
RMSD from standard bond length/angles (Å/°)	0.01/1.47
Average B value (Å^2^)	
Protein	26 (2395 atoms)
Waters	38 (284 atoms)
Chain B (Ligands)	45 (21 atoms)
Chain C (sugar molecules)	40 (107 atoms)
Ramachandran statistics (%)	
Favoured	98
Allowed	2
Outliers	0

* Figures in parentheses are for the highest resolution shell

## Results and discussion

We have purified pure cationic sorghum peroxidase from the Nigerian Sorghum grain variety SK 5912. The purification profile, using cation exchange chromatography, revealed a single basic peroxidase, similar to that previously reported for a related sorghum peroxidase [5]. SK 5912 sorghum peroxidase shows a high degree of sequence identity to other class III plant peroxidases, Figure S1.

**Fig. 1:**
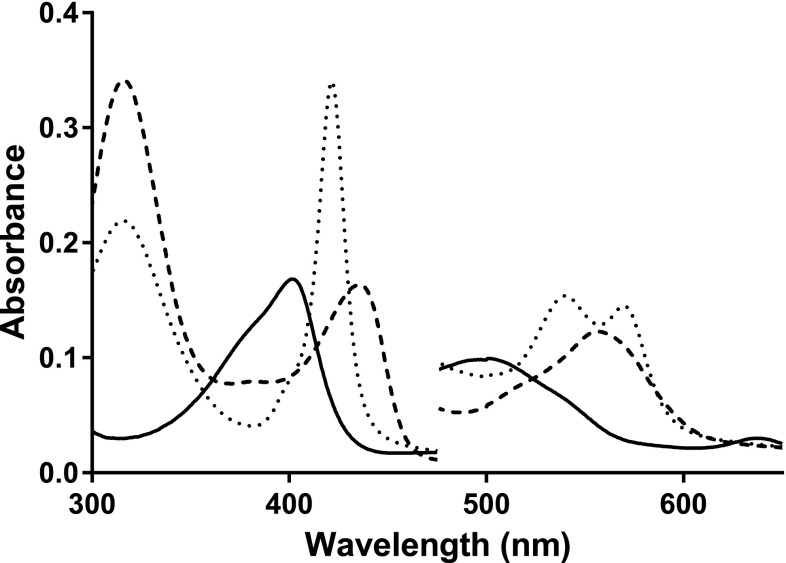
Electronic absorption spectra of SK 5912 sorghum peroxidase, showing the ferric (*solid line*), ferrous (*dashed line*) and ferrous-CO (*dotted line*) derivatives. Absorbance values in the visible region have been multiplied by a factor of 5 (100 mM sodium phosphate, pH 6.0, 25.0 °C)

**Fig. 2:**
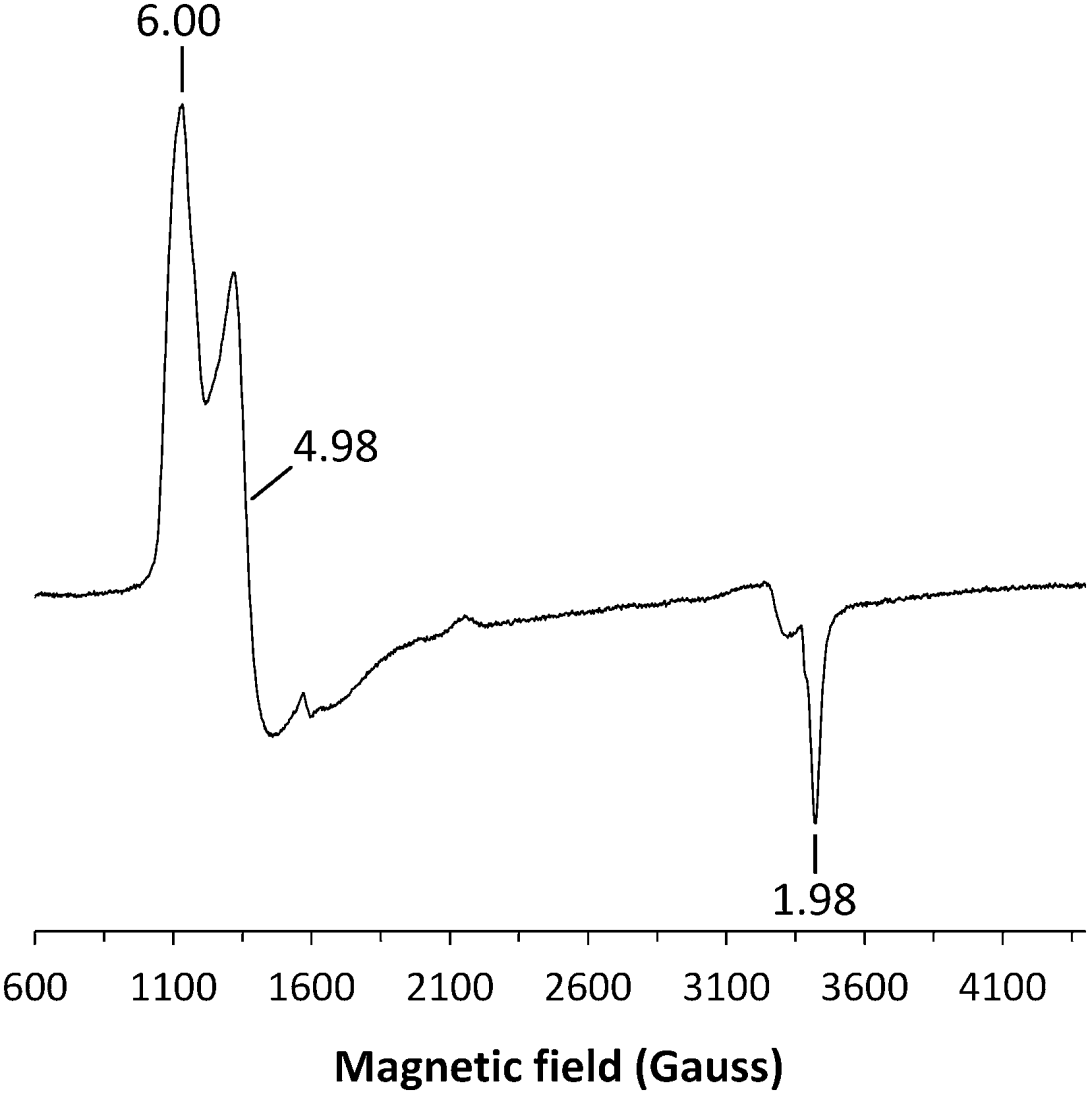
Low temperature EPR spectra of SK 5912 sorghum peroxidase (ca. 100 μM; the spectrum is given at a relative amplification of *G* = 0.50). The spectra were recorded at 10 K at the following instrumental conditions: microwave frequency *ν*_MW_ = 9.467 GHz, microwave power *P*_MW_ = 3.19 mW, modulation frequency *ν*_m_ = 100 kHz, modulation amplitude *A*_m_ = 5 G, time constant *τ* = 81.9 ms, scan rate *V* = 22.65 G/s. Conditions: pH = 6.0 (50 mM sodium phosphate)

UV–visible spectra of SK 5912 sorghum peroxidase (*λ*_max_ = 402 (*ε*_402_ = 119 mM^−1^ cm^−1^), 498, 638 nm, Fig. 1) are consistent with the presence of a predominantly high-spin heme. The spectrum of the ferric enzyme is independent of pH in the range 4.5–8.0, both in the presence and absence of 1 mM Ca^2+^ (see below). In agreement with that, the EPR spectra of the enzyme (Fig. 2) shows high-spin ferric heme signals (*g* = 6.00, 4.98 and 1.98) consistent with those typically observed in other peroxidases [19]. The spectrum of the ferric enzyme did not change in the presence of 1 mM Ca^2+^. The ferric enzyme binds cyanide (*K*_D_ = 12 ± 0.6 μM) to give a low-spin species (*λ*_max_ = 418, 538 nm, data not shown); the enzyme also binds azide (*K*_D_ = 19 ± 3 μM) to give a low-spin species (*λ*_max_ = 405 nm, data not shown). Anaerobic reduction of the ferric enzyme with dithionite results in a red-shift in the Soret band (from 402 to 436 nm) and a new peak at 556 nm, consistent with ferrous heme; the ferrous-CO complex (*λ*_max_ = 421, 540 and 570 nm) also forms normally, Fig. 1.

**Fig. 3:**
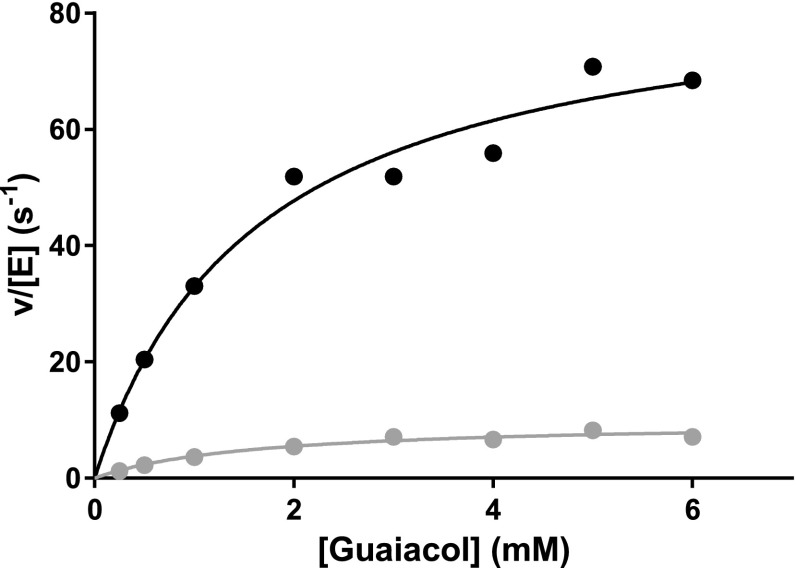
Steady state oxidation of guaiacol by SK 5912 sorghum peroxidase in the absence (*grey dot*) and presence (*black dot*) of Ca^2+^ (0.5 mM). Reaction conditions: 50 mM sodium acetate, pH 5.5, 25.0 °C. The *solid lines* are a fit to the Michaelis-Menten equation

The activity of SK 5912 sorghum peroxidase with guaiacol was measured under steady state conditions (*K*_M_ = 1.6 ± 0.4 mM, *k*_cat_ = 9.9 s^−1^); this activity was almost 10-fold higher in the presence of 0.5 mM CaCl_2_ although the *K*_M_ remained unaffected (*K*_M_ = 1.63 ± 0.4 mM, *k*_cat_ = 87 s^−1^, Fig. 3). This uplift in activity in the presence of calcium is consistent with the stopped-flow data and the crystallography, as explained below.

The potentiometric reduction of SK 5912 sorghum peroxidase (data not shown) yields a value for the Fe^3+^/Fe^2+^ reduction potential, *E*^*o*^ = −266 ± 5 mV. Values for the Fe^3+^/Fe^2+^ reduction potentials vary considerably across peroxidases (from around −30 to −320 mV), as summarised in a recent review [20]; the sorghum potential measured in this work falls in the range of those that have been measured for other peroxidases.

**Fig. 4:**
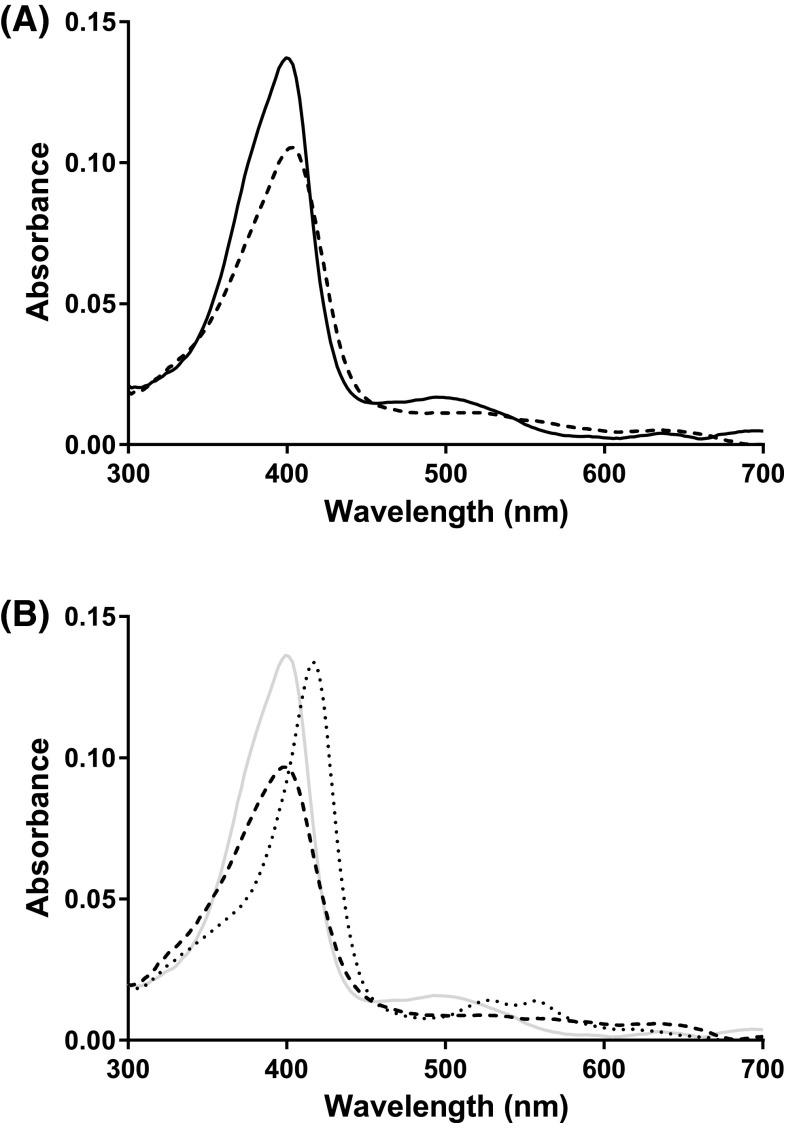
Reaction of SK5912 sorghum peroxidase with H_2_O_2_ monitored by stopped-flow photodiode array spectroscopy. **a** Deconvoluted spectra obtained from global analysis of the time-dependent spectral changes on mixing 1.7 μM enzyme with 50 μM H_2_O_2_ (time base of 50 s). The data were fitted to a one-step model (A → B, where A = ferric [*solid line*, collected at *t* = 1.28 ms) and B = Compound I (*dashed line*)] and the rate constant obtained from the global fitting is 0.19 s^−1^. **b** Deconvoluted spectra obtained from global analysis of the time-dependent spectral changes on mixing 1.7 μM enzyme with 50 μM H_2_O_2_ in the presence of 1 mM CaCl_2_ (time base of 50 s). The data were fitted to a one-step model (A → B, where A = Compound I (*dashed line*, collected at *t* = 1.28 ms) and B = Compound II (*dotted line*); for reference, the starting ferric spectrum (*grey*, at *t* = 0) is also shown) and the rate constant obtained from the global fitting is 0.1 s^−1^. Reaction conditions: 50 mM sodium acetate, pH 5.5, 25.0 °C

Figure 4 shows the spectral changes on reaction of SK 5912 sorghum peroxidase with H_2_O_2_. In the absence of Ca^2+^, Fig. 4a, Compound I forms slowly, so that the first spectrum observed (at 1.28 ms after mixing) is that of the ferric enzyme. This species converts slowly over 20 s to an intermediate that is consistent with formation of Compound I [*λ*_max_ = 405, 549(sh) and 636 nm]. The first order rate constant for ferric to Compound I conversion (0.19 s^−1^) is very slow compared with other peroxidases, which typically react rapidly with H_2_O_2_ with *k*_H2O2_ usually around 10^7^ M^−1^s^−1^ [21]. In the absence of Ca^2+^, the spectrum of Compound I is stable over short timescales (≈60 s) and we saw no evidence for conversion of Compound I to a Compound II species, as is typically observed for peroxidases; over longer periods (500 s, not shown) the spectrum of Compound I changes slightly and the Soret band shifts to 408 nm, and there is a slight reduction in the intensity of the peaks in the visible region, but no evidence for Compound II formation. Sorghum peroxidase is known to be activated by calcium ions (as noted in [5] and in our steady state data above) and therefore the stopped-flow experiment was repeated in the presence of 1 mM Ca^2+^, Fig. 4b. In this case, the reaction is much faster and occurs within the dead time of the instrument, so that the first observable species is Compound I (and not the ferric enzyme, as above).^1^ In contrast to the behaviour in the absence of Ca^2+^, decay of Compound I leads to clean formation of a Compound II species (*λ*_max_ = 415, 526 and 556 nm) with a rate constant of ≈0.1 s^−1^ (Fig. 4b).

**Fig. 5:**
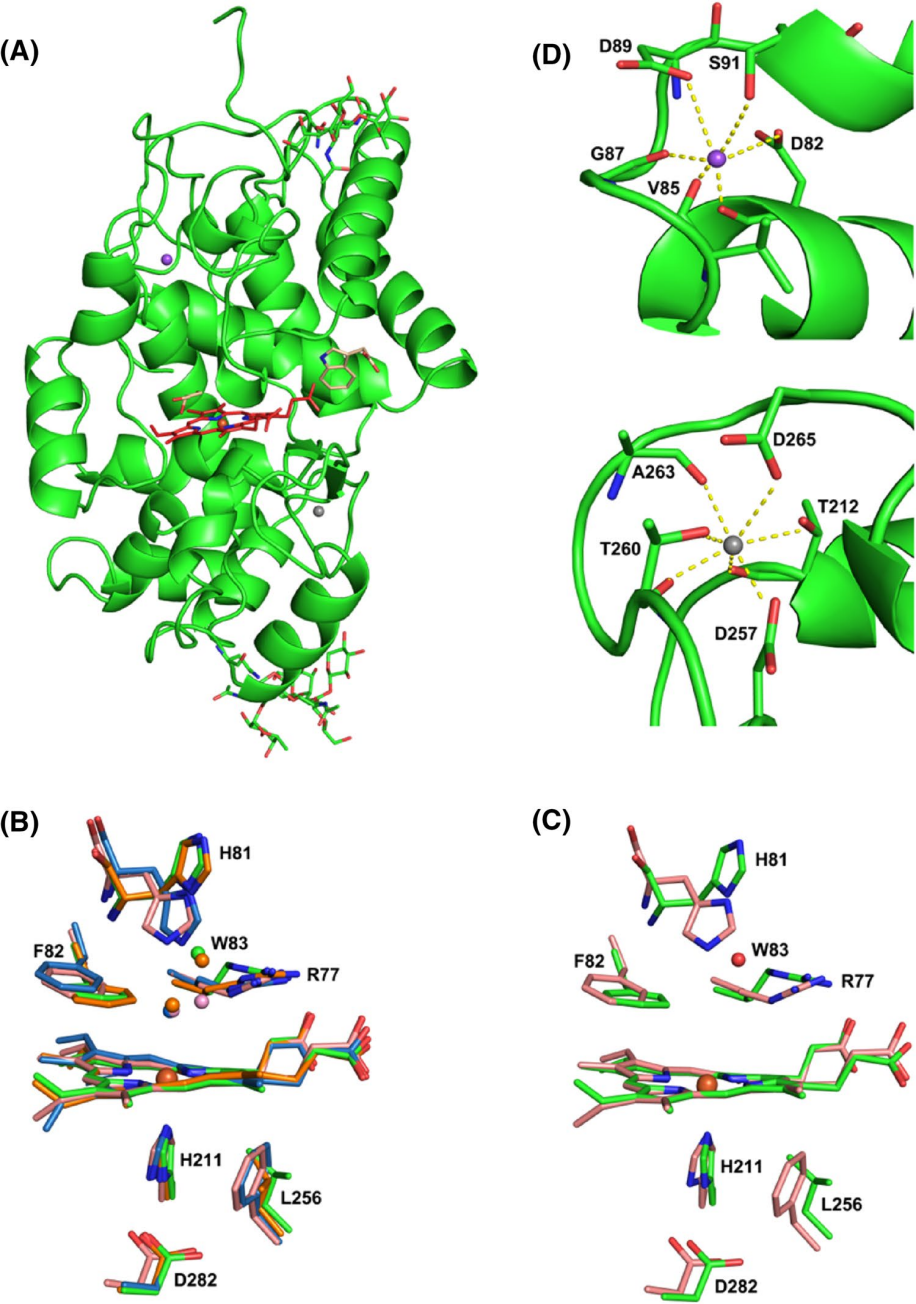
**a** The overall structure of SK5912 sorghum peroxidase, showing the heme (in *red*), glycosylation sites (in *green*), IAA and glycerol (in *beige*), sodium ion (in *purple*) and calcium ion (in *grey*). **b** Superposition of the active sites of four class III peroxidases: sorghum peroxidase (*green*), HRP (*pink*), BP1 (*orange*), PNP (*blue*). The numbering corresponds to residues in sorghum peroxidase. Water molecules are coloured in the same colour as the corresponding protein molecules. BP1 and sorghum peroxidase have a reorientated distal histidine and a structural water molecule (W83 in the sorghum enzyme). **c** Superposition of the active sites of sorghum peroxidase (*green*) and HRP (*pink*). W83 belongs to the sorghum enzyme. The overlay shows the distance between ND1 of distal histidine of sorghum peroxidase to Fe is much further (8.16 Å) than that of HRP (5.9 Å), due to the displacement of distal histidine in the sorghum peroxidase. **d** *Top* The distal sodium site. The sodium ion is shown as *purple sphere* and metal–ligand bonds are shown as *dashed yellow lines*. *Bottom* the proximal calcium site. The calcium ion is shown as *grey sphere* and metal–ligand bonds are shown as *dashed yellow lines*

The structure of SK 5912 sorghum peroxidase is presented in Fig. 5a. The overall structure contains 306 residues and closely resembles the structures of other class III peroxidases—namely barley peroxidase (BP1 [16]), with an RMSD of 0.652 over 306 Cα atoms, as well as peanut peroxidase (PNP) [22] and horseradish peroxidase (HRP) [23]. The class III heme peroxidases share common structural features, including four conserved disulphide bridges which are also observed in the sorghum enzyme (C50–C131, C83–C88, C138–C333 and C218–C245). Two glycosylation sites are found, which are positioned in the loop regions that are solvent accessible and exterior to the core helical structure. The active sites of the four class III enzymes are compared in Figs. 5b and S2. A notable difference between the typical class III peroxidases (HRP, PNP) and the sorghum enzyme is the position of the distal histidine: His 81 in the sorghum enzyme is much further from the iron (Nδ1 to Fe distance = 8.16 Å, compared to 5.9 Å in HRP, Fig. 5c). The loop between Asp 95 and Pro 114 is displaced (not shown), which may have allowed movement of the histidine side chain. In the sorghum structure, a water molecule (W83, Fig. 5c) is found in the region that is occupied by the distal histidine in both HRP and PNP, and this water forms a hydrogen bond (2.75 Å) to Nδ1 of His81. A movement of the distal histidine is also observed in the BP1 structure [16], Fig. 5b. Barley peroxidase does not form Compound I under the conditions (pH 5.5–8.5) used for its crystallisation, and it was proposed [16] that the reorientation of the histidine was responsible for the inactivity. We note that the Cα positions of the distal histidines in the sorghum and HRP structures are very similar (0.74 Å), Fig. 5c, which may indicate that the presence of W83 pushes the His side chain away from the heme so that reaction with peroxide only occurs in the absence of this water molecule and once the His side chain swings back towards the iron as in other peroxidases.

**Fig. 6:**
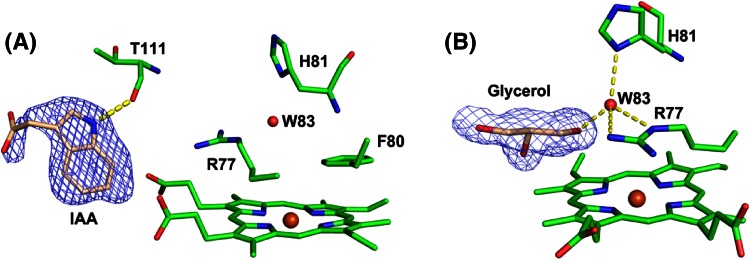
Substrate binding in sorghum peroxidase. **a** Figure showing binding of IAA at the γ-heme edge. The nitrogen atom of IAA makes a hydrogen bond to the carbonyl oxygen of Thr111 (2.75 Å). **b** Hydrogen bonding structure around W83, and binding of glycerol at the δ-heme edge. W83 makes hydrogen bonds to ND1 of His 81 (2.76 Å), NE (2.79 Å) and NH2 (3.0 Å) of Arg 77, and oxygen atom of glycerol (2.7 Å). In both figures, the 2F_o_-F_c_ map is contoured at 1σ

Close to the active site there is a region of density at the γ-heme edge, Fig. 6a, which is in the same location as that known to be used for binding of other substrates in heme peroxidases (e.g. ascorbate in ascorbate peroxidase, and Mn^2+^ in manganese peroxidase [24]^2^ ). This density is consistent with the presence of indole-3-acetic acid (IAA). IAA is a major plant hormone, and is synthesised biosynthetically from tryptophan [25]. IAA is a well documented substrate for HRP [21], and is reported as a substrate for sorghum peroxidase [5]. Since the SK 5912 sorghum peroxidase is isolated from source (i.e. directly from the sorghum grains), we suggest that this IAA binds to the enzyme and is carried through the isolation and purification process. Binding of such a substrate at the γ-heme edge in the sorghum structure is thus consistent with patterns of substrate binding behaviour across the wider family of heme peroxidases.

The class III heme peroxidases typically contain two calcium binding sites. In the sorghum structure there are also two metal ions, and at the same locations as found in HRP and PNP, Fig. 5a, d; in the sorghum structure, these are assigned as Na^+^ (on the distal side) and Ca^2+^ (on the proximal side) binding sites based on the ligand coordination geometries. The proximal metal site is seven coordinate, Fig. 5d (bottom panel), with an average distance of 2.40 Å to its ligands T212 (O and OG1), D257 (OD2), T260 (Oand OG1), A263 (O) and D265 (OD1). It was refined as a Ca^2+^ based on the bond distance to the ligands and the coordination number. This proximal Ca^2+^ site is indirectly connected to the proximal histidine residue (His211) through the adjacent residue Thr 212. The second (distal) metal binding site is observed to be six-coordinate in the sorghum structure, Fig. 5d, and with longer bond lengths to the ligands [D82 (O and OD1), V85 (O), G87 (O), D89 (OD1) and S91 (OG); average distance 2.44 Å], which is consistent with the presence of a Na^+^ ion; this is different from the HRP and PNP structures, which show seven coordinate Ca^2+^ in the same position. This site was, thus refined as sodium, as also assigned in the BP1 structure,^3^ but the presence of sodium at this site instead of the more typical calcium might merely be a reflection of the crystallization conditions (in sodium formate). The presence of Ca^2+^ and Na^+^ ions at these sites is consistent with the Ca^2+^-dependence of the steady state and pre-steady state kinetics above. It is probably the case that the catalytic activity of the sorghum enzyme is dependent on metal ion incorporation at these positions, or even that the activity is dependent on replacement of the distal sodium site with calcium (which would account for the calcium activation). Calcium incorporation at the distal metal site might even be accompanied by structural changes that shorten the distance of the distal histidine from the heme iron. Previous work would be consistent with this interpretation, as Smith and co-workers observe [26] that Ca^2+^ is required for formation of fully folded HRP during recombinant expression of HRP in *E. coli*, and they [23] and others [27] have noted that calcium depletion is likely to affect catalytic performance in HRP. BP1 is similarly activated by calcium [28]. The structural and kinetic information presented in this work for the sorghum enzyme are consistent with these studies on the other class III enzymes, and our data also align nicely with the earlier suggestion [5] that the activity of sorghum enzymes is calcium dependent.

## Electronic supplementary material

Supplementary material 1 (PDF 1036 kb)

## Footnotes

1 Reducing the temperature at which the reaction was carried out (to 10 °C), or decreasing the concentration of Ca^2+^ (to 100 μM), did not slow down the rate at which Compound I was formed, so that ferric enzyme was never observed as the first isolated spectrum in the presence of Ca^2+^.

2 There is another region of density, close to the δ-heme edge, which we have assigned as glycerol (probably binding to the enzyme during the purification process), Fig. 6b.

3 Refining the distal metal ion as Ca^2+^ gave a negative peak in F_o_-F_c_ map. The B factor of the Ca^2+^ also doubled (~30 Å^2^) when the metal was refined as Ca^2+^, which was also twice the B factor of its surrounding ligands. This was not the case when metal was refined at Na^+^, as the B factors of the metal and the ligands in this case were lower and similar in magnitude to one another.

## References

[1] Paterson AH, Bowers JE, Bruggmann R, Dubchak I, Grimwood J, Gundlach H, Haberer G, Hellsten U, Mitros T, Poliakov A, Schmutz J, Spannagl M, Tang H, Wang X, Wicker T, Bharti AK, Chapman J, Feltus FA, Gowik U, Grigoriev IV, Lyons E, Maher CA, Martis M, Narechania A, Otillar RP, Penning BW, Salamov AA, Wang Y, Zhang L, Carpita NC, Freeling M, Gingle AR, Hash CT, Keller B, Klein P, Kresovich S, McCann MC, Ming R, Peterson DG, Mehboob-ur R, Ware D, Westhoff P, Mayer KF, Messing J, Rokhsar DS (2009). The *Sorghum bicolor* genome and the diversification of grasses. Nature.

[2] Sae SW, Kadoum AM, Cunningh BA (1971). Purification and some properties of Sorghum grain esterase and peroxidase. Phytochemistry.

[3] Eze SO, Chilaka FC, Nwanguma BC (2000). Purification and characterization of sorghum (KSV 8) peroxidase. Plant Products Resour J.

[4] Omidiji O, Okpuzor J, Otubu O (2002). Peroxidase activity of germinating *Sorghum bicolor* grains: effect of some cations. J Sci Food Agr.

[5] Dicko MH, Gruppen H, Hilhorst R, Voragen AG, van Berkel WJ (2006). Biochemical characterization of the major sorghum grain peroxidase. FEBS J.

[6] Ogbonna AC, Obi SKC, Okolo BN (2003). Protein modification in malting sorghum. World J Microb Biotechnol.

[7] Nwanguma BC, Eze MO (1995). Heat sensitivity, optimum ph and changes in activity of Sorghum peroxidase during malting and mashing. J I Brewing.

[8] Antonini M, Brunori E (1971). Hemoglobin and myoglobin and their reactions with ligands.

[9] Patel N, Jones DK, Raven EL (2000). Investigation of the haem-nicotinate interaction in leghaemoglobin. Role of hydrogen bonding. Eur J Biochem.

[10] Efimov I, Parkin G, Millett ES, Glenday J, Chan CK, Weedon H, Randhawa H, Basran J, Raven EL (2014). A simple method for the determination of reduction potentials in heme proteins. FEBS Lett.

[11] Clark WM (1972). Oxidation-reduction potentials of organic systems.

[12] Efimov I, Papadopoulou ND, McLean KJ, Badyal SK, Macdonald IK, Munro AW, Moody PC, Raven EL (2007). The redox properties of ascorbate peroxidase. Biochemistry.

[13] Kabsch W (2010). XDS. Acta Crystallogr D Biol Crystallogr.

[14] Collaborative Computational Project, N (1994). The CCP4 suite: programs for protein crystallography. Acta Crystallogr.

[15] McCoy AJ, Grosse-Kunstleve RW, Adams PD, Winn MD, Storoni LC, Read RJ (2007). Phaser crystallographic software. J Appl Crystallogr.

[16] Henriksen A, Welinder KG, Gajhede M (1998). Structure of barley grain peroxidase refined at 1.9-A resolution. A plant peroxidase reversibly inactivated at neutral pH. J Biol Chem.

[17] Murshudov GN, Vagin AA, Dodson EJ (1997). Refinement of macromolecular structures by the maximum-likelihood method. Acta Crystallogr Sect D.

[18] Agirre J, Davies G, Wilson K, Cowtan K (2015). Carbohydrate anomalies in the PDB. Nat Chem Biol.

[19] Jones DK, Dalton DA, Rosell FI, Raven EL (1998). Class I heme peroxidases: characterization of soybean ascorbate peroxidase. Arch Biochem Biophys.

[20] Battistuzzi G, Bellei M, Bortolotti CA, Sola M (2010). Redox properties of heme peroxidases. Arch Biochem Biophys.

[21] Dunford HB (1999). Heme peroxidases.

[22] Schuller DJ, Ban N, Huystee RB, McPherson A, Poulos TL (1996). The crystal structure of peanut peroxidase. Structure.

[23] Gajhede M, Schuller DJ, Henriksen A, Smith AT, Poulos TL (1997). Crystal structure of horseradish peroxidase C at 2.15 A resolution. Nat Struct Biol.

[24] Gumiero A, Murphy EJ, Metcalfe CL, Moody PCE, Raven EL (2010). An analysis of substrate binding interactions in the heme peroxidase enzymes: a structural perspective. Arch Biochem Biophys.

[25] Celenza J (2001). Metabolism of tyrosine and tryptophan—new genes for old pathways. Curr Opin Plant Biol.

[26] Smith AT, Santama N, Dacey S, Edwards M, Bray RC, Thorneley RN, Burke JF (1990). Expression of a synthetic gene for horseradish peroxidase C in *Escherichia coli* and folding and activation of the recombinant enzyme with Ca^2+^ and heme. J Biol Chem.

[27] Shiro Y, Kurono M, Morishima I (1986). Presence of endogenous calcium ion and its functional and structural regulation in horseradish peroxidase. J Biol Chem.

[28] Rasmussen CB, Hiner AN, Smith AT, Welinder KG (1998). Effect of calcium, other ions, and pH on the reactions of barley peroxidase with hydrogen peroxide and fluoride. Control of activity through conformational change. J Biol Chem.

