# Supplementary material for: Structural and spectroscopic characterisation of a heme peroxidase from sorghum
Source: J Biol Inorg Chem. 2015 Dec 14;21:63–70. doi: 10.1007/s00775-015-1313-z (PMC4771821; doi:10.1007/s00775-015-1313-z)
Supplement: Supplementary file 1 — Supplementary material 1 (PDF 1036 kb) [file 775_2015_1313_MOESM1_ESM.pdf]

Structural and spectroscopic characterisation of a heme peroxidase from sorghum

Chukwudi I. Nnamchi[1], Gary Parkin[2], Igor Efimov[2], Jaswir Basran[3], Hanna Kwon[3], Dimitri A. Svistunenko[4], Jon Agirre,[5] Bartholomew N. Okolo[1], Anene Moneke[1], Bennett C. Nwanguma[6], Peter C. E. Moody[3], Emma L. Raven[2,*]

[1] *Department of Microbiology, University of Nigeria, Nsukka*
[2] *Department of Chemistry, University of Leicester, University Road, Leicester, LE1 7RH, UK*
[3] *Department of Molecular and Cell Biology, Henry Wellcome Laboratory for Structural Biology, University of Leicester, Lancaster Road, Leicester, LE1 9HN, UK*
[4] *School of Biological Sciences, University of Essex, Wivenhoe Park, Colchester CO4 3SQ, UK*
[5] *York Structural Biology Laboratory, Department of Chemistry, The University of York, Heslington, York, YO10 5DD, UK*
[6] *Department of Biochemistry, University of Nigeria, Nsukka*

Figure S1. Sequence alignment of sorghum peroxidase, BP1, HRP and PNP. The sorghum peroxidase has the highest sequence identity to BP1 (69 %) then to PNP (45 %) and HRP (44 %). The figure was generated by ESPript (1).

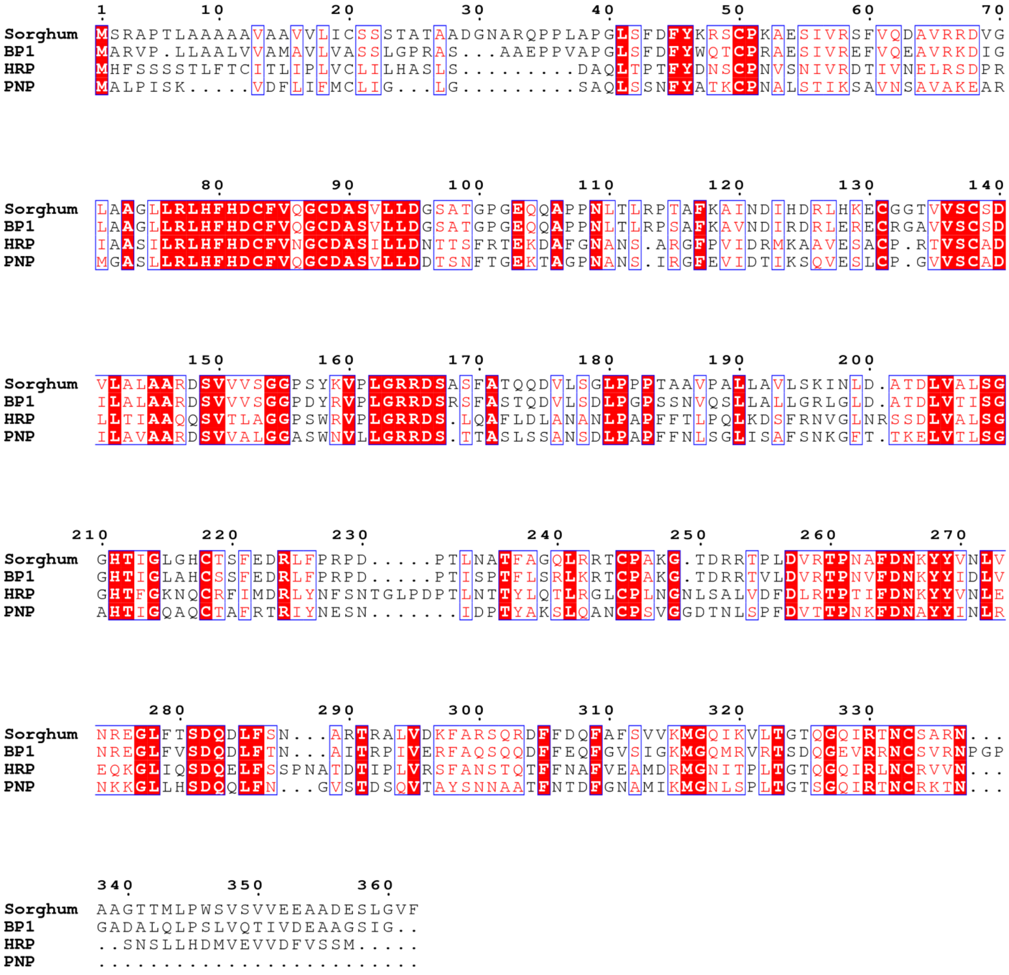

Figure S2. Comparison of the active sites of (A) sorghum peroxidase, (B) HRP, (C) BP1 and (D) PNP. The sorghum peroxidase is the only structure with 5-coordinated heme. The sorghum and BP1 enzymes shares some structural characteristics such as re-orientation of the distal histidine, presence of structural water molecules (W83 in sorghum and W626 in BP1) and the presence of a leucine residue in the proximal heme pocket (instead of phenylalanine residues found in HRP and PNP). Water molecules are shown as red spheres.

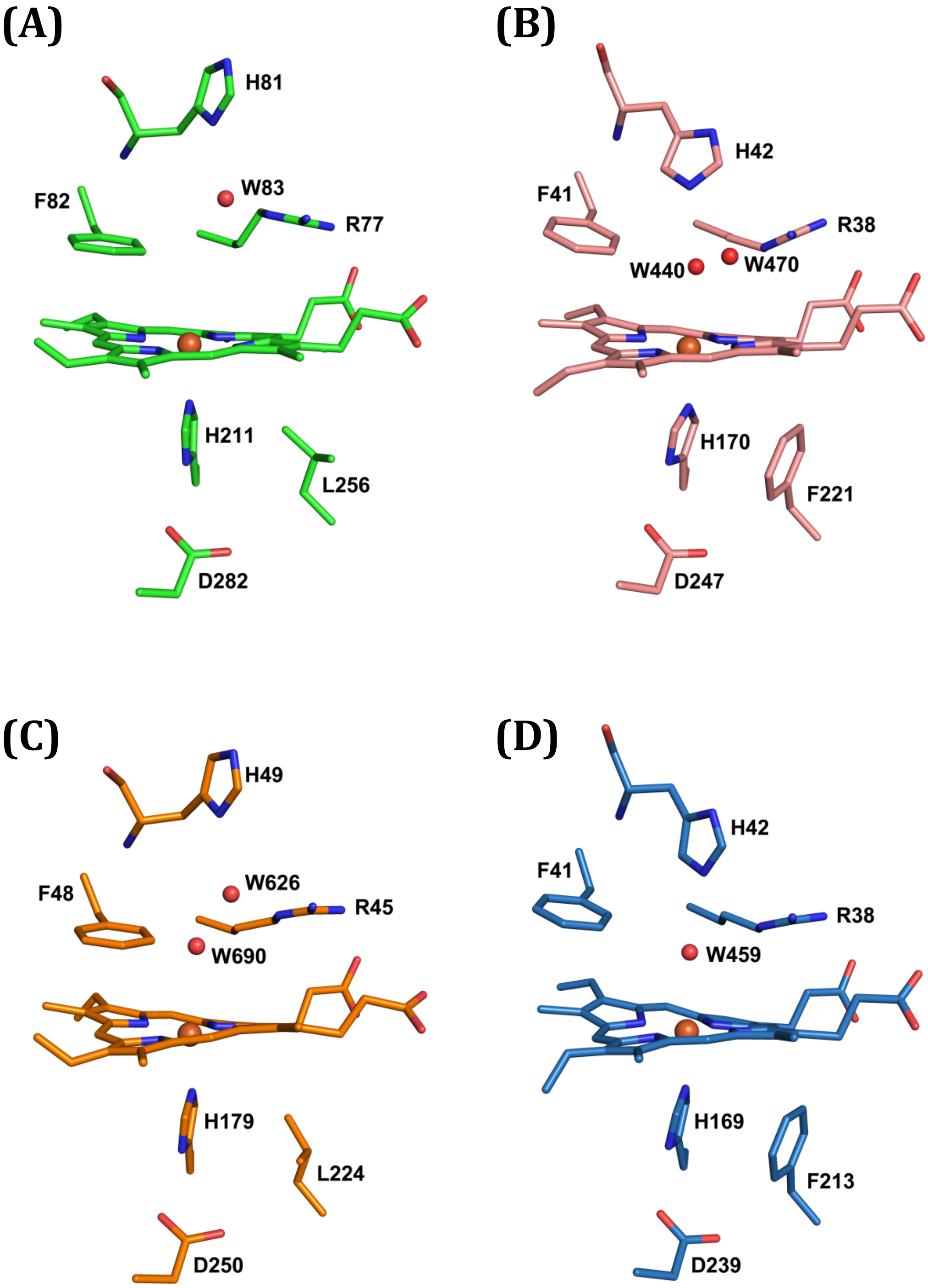

1. Gouet P, Robert X, & Courcelle E (2003) ESPript/ENDscript: Extracting and rendering sequence and 3D information from atomic structures of proteins. *Nucleic acids research* 31(13):3320-3323.
